# Improved count rate corrections for highest data quality with PILATUS detectors

**DOI:** 10.1107/S0909049512003950

**Published:** 2012-03-16

**Authors:** P. Trueb, B. A. Sobott, R. Schnyder, T. Loeliger, M. Schneebeli, M. Kobas, R. P. Rassool, D. J. Peake, C. Broennimann

**Affiliations:** a DECTRIS Ltd, 5400 Baden, Switzerland; b School of Physics, The University of Melbourne, Victoria 3010, Australia

**Keywords:** PILATUS detectors, photon counting, count rate correction, bunch pattern, Monte Carlo simulation

## Abstract

The PILATUS detector system is widely used for X-ray experiments at third-generation synchrotrons. It is based on a hybrid technology combining a pixelated silicon sensor with a CMOS readout chip. Its single-photon-counting capability ensures precise and noise-free measurements. The counting mechanism introduces a short dead-time after each hit, which becomes significant for rates above 10^6^ photons s^−1^ pixel^−1^. The resulting loss in the number of counted photons is corrected for by applying corresponding rate correction factors. This article presents the results of a Monte Carlo simulation which computes the correction factors taking into account the detector settings as well as the time structure of the X-ray beam at the synchrotron. The results of the simulation show good agreement with experimentally determined correction factors for various detector settings at different synchrotrons. The application of accurate rate correction factors improves the X-ray data quality acquired at high photon fluxes. Furthermore, it is shown that the use of fast detector settings in combination with an optimized time structure of the X-ray beam allows for measurements up to rates of 10^7^ photons s^−1^ pixel^−1^.

## Abstract 2

A Monte Carlo simulation is presented, which computes the rate correction factors taking into account the detector settings and the time structure of the X-ray beam. The results show good agreement with experimentally determined correction factors.

## 1. Introduction

X-ray crystallography profits greatly from the usage of hybrid pixel detectors, which allow for high dynamic range, low noise performance, high frame rates, as well as radiation tolerance. These detectors, which were originally developed for high-energy physics, combine pixelated silicon sensors with CMOS-based readout technology. Their ability to exactly measure the number of photons guarantees for very precise data, but also introduces some dead-time during which the pixel is insensitive for incoming photons. For the PILATUS detector, this dead-time is of the order of some hundred nanoseconds, which becomes significant when the incoming photon rate exceeds 10^6^ counts s^−1^ (cps). As a consequence the observed number of photons will be lower than the actual number of incoming photons. Fortunately this effect can be corrected for by applying rate correction factors unless the incoming photon rate is too high.

Assuming a paralysable detector model, the rate correction factors can be derived analytically if the time interval between two photon bunches is constant. For more complicated synchrotron time structures there exist no analytical solutions. In these cases Monte Carlo simulations can be used to derive the correction factors as explained by Bateman (2000 ▶). Contrary to its claims, the model presented in that publication is not a general solution for ‘any given detector system when used with the typical beam structures encountered in a synchrotron radiation source’ (cited from Bateman, 2000 ▶). On the one hand it only considers a limited number of time structures; on the other hand it uses an idealized detector model. A detector like the PILATUS system cannot be characterized by means of a single number (the detector dead-time). The behaviour of its readout chip during an acquisition is highly dynamic, involving non-linear pile-up in the amplifier or voltage shifts owing to high activity. To take these effects into account, a detailed circuit simulation of the readout chip is required.

## 2. The PILATUS detector system

**Figure 1:**
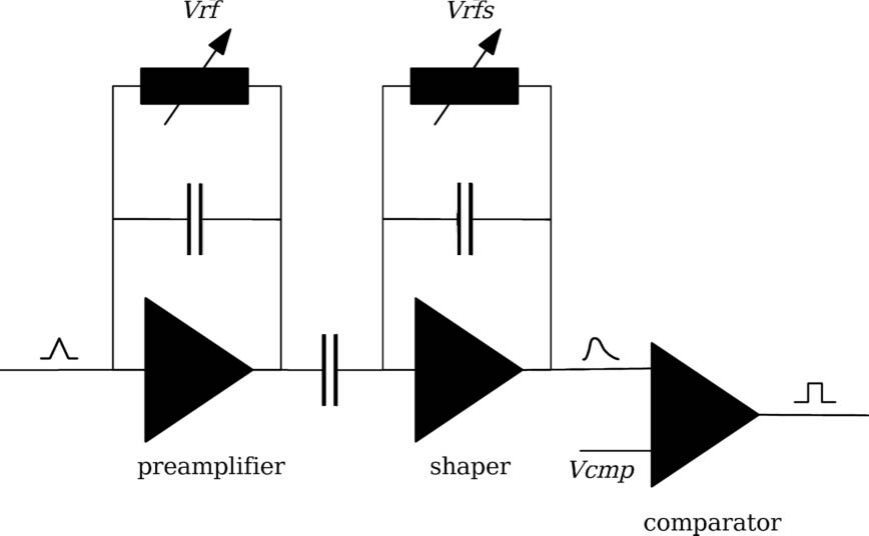
Schematic of the simulated pixel circuit. *Vrf*, *Vrfs* and *Vcmp* are configurable control voltages.

The PILATUS system basically consists of a silicon sensor, which is electrically connected to a CMOS readout chip. The absorption of the X-ray photons in the silicon leads to the creation of electron–hole pairs. The charge generated in this way is collected at the bottom of the sensor with the help of an applied high-voltage electric field. Each sensor pixel is electrically connected to a cell of the readout chip, which is responsible for the amplification and registration of the signals. A simplified schematic of the readout unit is shown in Fig. 1 ▶. The collected charge is first amplified by the charge-sensitive preamplifier. The gain of this preamplifier can be adjusted by a control voltage called *Vrf*. Subsequently the signal is formed by the shaper stage, whose control voltage *Vrfs* is usually kept fixed. The output of the shaper is then compared with an adjustable threshold voltage *Vcmp*. As long as the shaper output exceeds the threshold level the comparator output remains at a high level. During that time the corresponding pixel is unable to register further photons, which leads to a limited count rate capability. Some important properties of the PILATUS detector are summarized in Table 1 ▶. More details can be found by Broennimann *et al.* (2006 ▶).

## 3. Simulation

The rate correction factors of the PILATUS pixel detector depend on the detector settings, such as the gain of the charge-sensitive preamplifier and the energy threshold, as well as on the energy and time structure of the X-ray beam. To determine the correction factors as a function of these parameters, a simulation of the data acquisition process was set up. The computation of the observed count rate for a given incoming photon rate proceeds along the following steps: (i) generating the temporal sequence of the incoming photons; (ii) deriving the charge collected by a pixel as a function of time; (iii) simulating the response of the readout chip to this charge. The details of these steps are explicated in the following paragraphs.

In this article the term ‘incoming rate’ will refer to the photon rate as it would be determined by the PILATUS detector in the absence of any counting loss due to high photon rates. Obviously the incoming rate is not identical to the physical photon flux on the area of a pixel. Owing to the incomplete photon absorption in the sensor as well as the suppression of photons with an energy lower than the threshold energy, the incoming rate is lower than the physical photon rate on a pixel. As a consequence of the applied definition, the observed rate of a pixel will always be equal to the incoming rate in the limit of low count rates.

### 3.1. Generating the incoming photon sequence

The temporal sequence of the incoming photon flux is defined by the bunch structure of the synchrotron. As an input to the simulation, the user has to specify the instant in time as well as the relative number of electrons for each bunch in a synchrotron cycle. The statistical precision of the predicted rate correction factors will depend on the duration of the simulated acquisition. The duration can be specified either by the minimal number of bunches and/or the minimal number of synchrotron cycles to be simulated.

First, the mean number of photons from each bunch is computed for the specified incoming photon rate. The computation ensures that the mean number of photons from each bunch is proportional to the relative number of electrons in the corresponding bunch and that the observed rate averaged over a complete synchrotron cycle is equal to the specified incoming rate in the limit of low count rates. Second, the actual number of photons from each bunch has to be determined by use of a random number generator. The random numbers are required to obey a Poisson statistics with a mean value equal to the mean number of photons of this bunch.

### 3.2. Deriving the charge collected by a pixel as a function of time

**Figure 2:**
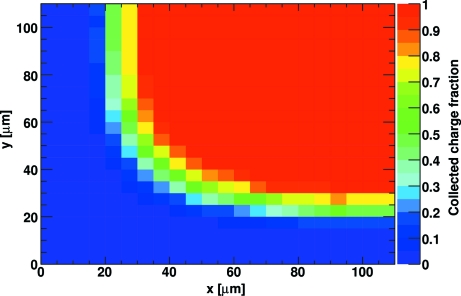
Collected charge fraction as a function of the photon impact position on a pixel. Shown is a quarter of a pixel (somewhat extended over the pixel borders) with the pixel centre in the upper right corner.

For a mono-energetic X-ray beam the (mean) charge generated in the sensor is proportional to the total photon energy in a bunch. The charge collected at the input of the readout chip depends on the impact point of the photon on the pixel surface. The charge of a photon impinging near the border of a pixel will be shared between several pixels, while the charge of a photon impinging in the centre of a pixel will be completely detected by one pixel. To take this effect into account, a random impact point on the sensor is selected for every photon assuming a uniform illumination of the pixel. This impact point is allowed to lie outside the pixel border because some small fraction of the charge will drift towards the pixel under investigation. The charge fraction detected by a pixel as a function of the impact position was taken from Schubert *et al.* (2010 ▶) and corrected for the experimental uncertainty to match the theoretical charge spread as computed by
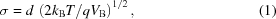
where *d* = 320 µm is the sensor thickness, *k*_B_ the Boltzmann constant, *T* the temperature, *q* the elementary charge and *V*_B_ the applied bias voltage (Lutz, 1997 ▶). For *V*_B_ = 150 V and *T* = 300 K, this yields a charge spread of 5.94 µm. Fig. 2 ▶ shows the collected charge fraction for this value as a function of the photon impact position on a quarter of a pixel. Experimental measurements with the PILATUS detector tend to give larger values for the charge spread than the theoretical value reported above. Since the reason for this discrepancy is unclear (Broennimann *et al.*, 2006 ▶), the theoretical value is used. It was verified that the results reported below do not significantly depend on the usage of the theoretical or the experimental value.

### 3.3. Simulating the response of the readout chip

The observed count rate is derived by a circuit simulation (Spectre) of the PILATUS pixel unit cell, which comprises the preamplifier, shaper and comparator circuits (*cf.* Fig. 1 ▶). The charge collected in the sensor is modelled as a current pulse connected to the preamplifier input. The observed count rate is finally determined from the number of pulses at the comparator output.

## 4. Validation

### 4.1. Gain dependency

The dead-time of the PILATUS detector is mainly determined by the preamplifier gain settings. A high gain results in a broader pulse, which paralyses the comparator for a longer time. Therefore the observed count rate will be lower for higher gain settings.

**Figure 3:**
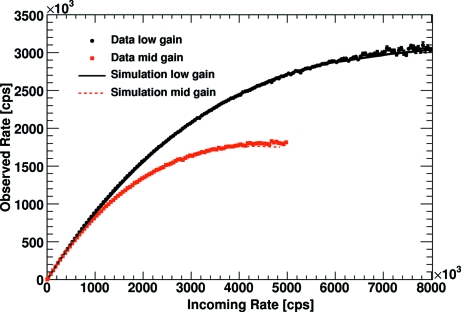
Comparison of the results of the Monte Carlo simulation with the experimental data taken at the SLS for different preamplifier gain settings.

To study the gain and threshold dependencies of the rate correction factors, several data sets were acquired at the Swiss Light Source (SLS) at the Paul Scherrer Institute in Villigen, Switzerland (Kraft *et al.*, 2009 ▶). The experimental parameters are summarized in Table 2 ▶. These data were reanalysed to validate the Monte Carlo simulation described above. It is found that the simulation tends to count faster than the actual readout chip. The particular reason for this deviation between simulation and experiment is currently not known. The difference can be corrected for by adding a constant offset of 34 mV to the gain control voltage *Vrf*. Regarding the complexity of the circuit simulation, this rather small deviation between the simulation and the actual readout chip is not surprising. After this correction the difference between the simulation and the experimental data (averaged over all investigated pixels) is around 2% in the high-count-rate region (>5 × 10^5^ counts s^−1^) (*cf.* Fig. 3 ▶). Correcting the data with the computed rate correction factors, the rate correction results in a systematic error of about 3% on the observed number of counts for an individual pixel for low gain settings and an incoming photon rate of 10^6^ counts s^−1^. For mid gain settings, the corresponding systematic error at 10^6^ counts s^−1^ is ∼4%.

### 4.2. Energy threshold dependency

**Figure 4:**
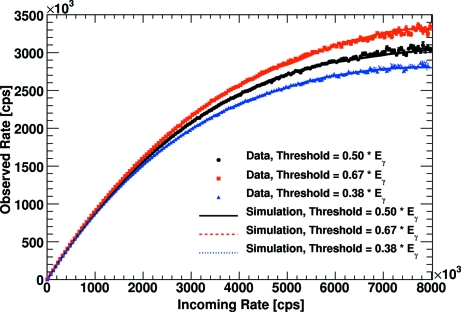
Comparison of the results of the Monte Carlo simulation with the experimental data taken at the SLS for different energy thresholds.

A higher energy threshold decreases the time during which the pulse is above the comparator threshold voltage. This decreases the detector dead-time and consequently increases the observed count rate. This effect is clearly observed in the SLS data. Fig. 4 ▶ shows the measured data in comparison with the predictions of the implemented simulation. The simulation perfectly predicts the observed count rate as a function of the energy threshold.

### 4.3. Bunch structure dependency

**Figure 5:**
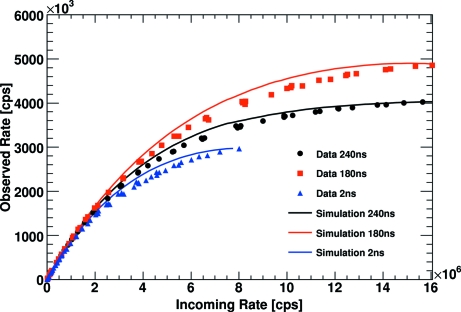
Comparison of the results of the Monte Carlo simulation with the experimental data taken at the AS for different beam time structures for low gain settings and an energy threshold at half the X-ray energy.

To study the dependency on the time structure of the X-ray beam, additional data sets were recorded at the Australian Synchrotron (AS) (Sobott *et al.*, 2012 ▶). To maximize the detector count rate capability, the time between two electron bunches was adjusted to be slightly larger than the detector dead-time. Given the time period of the synchrotron of 720 ns, the three- and four-bunch modes result in bunch separations of 240 ns and 180 ns. For comparison, a reference data set was taken in the default bunch mode with 2 ns bunch spacing, whose current follows a trapezoidal shape as a function of time. Fig. 5 ▶ shows that the optimized time structures allow for data taking up to an incoming rate of 10^7^ photons s^−1^ pixel^−1^. The experimental parameters are summarized in Table 3 ▶. The Monte Carlo simulation is able to predict the observed count rate for the completely different time structure at the Australian Synchrotron with an accuracy of better than 2.5% for incoming rates below 10^6^ counts s^−1^ and an accuracy of better than 7% over the whole range. An important difference to the SLS data set is the fact that the AS data are based on only one pixel. This might explain the larger deviation from the simulation, which reproduces the behaviour of an average pixel. From the SLS data it is known that the dead-time of different pixels varies of the order of 10%.

An important lesson from the AS data concerns the interplay of the time structure of the beam and the detector settings. Fig. 5 ▶ shows that a bunch separation of 180 ns together with low gain settings allows for measurements up to an incoming rate of 10^7^ photons s^−1^ pixel^−1^. This is much higher than for other beam time structures as explained in the following section.

## 5. Results

In this section the results of the implemented simulation for different synchrotron time structures are presented.

**Figure 6:**
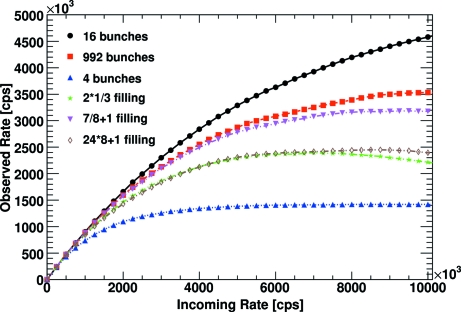
Comparison of different operating modes of the ESRF synchrotron for low gain settings and an energy threshold at half the X-ray energy.

An interesting synchrotron with many different time structures of the X-ray beam is the European Synchrotron Radiation Facility (ESRF) in Grenoble, France. Fig. 6 ▶ shows the observed count rates for the PILATUS detector for different operating modes. There are two modes, labelled ‘992 bunches’ and ‘7/8+1 filling’ for which the PILATUS detector behaves as if it sees a continuous X-ray beam. The modes ‘2*1/3 filling’ and ‘24*8+1 filling’ include some gaps in the X-ray flux. Compared with the continuous case, the photons are squeezed to shorter intervals resulting in more pile-up and therefore lower count rates for the same incoming rates. The favourable mode with 16 bunches per circulation period has a bunch separation of 176 ns, which is almost identical to the AS mode with 180 ns bunch spacing described above. The most disadvantageous mode involves only four bunches separated by 704 ns. The observed rate saturates at a value of 1/704 ns = 1.42 × 10^6^ counts s^−1^. It is important to note that this value is only determined by the X-ray time structure and is independent of the detector settings. Even a counting detector with lower dead-time will show the same saturation, since it cannot resolve the number of photons inside a bunch. A better performance can only be achieved by a detector that is able to determine the number of photons from each bunch.

**Figure 7:**
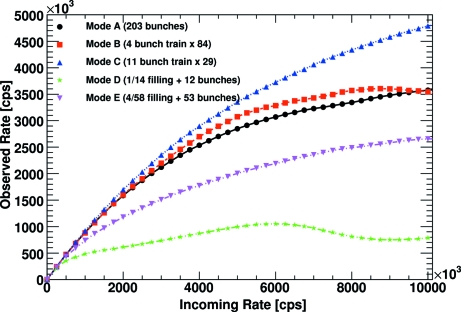
Comparison of different operating modes of the SPring-8 synchrotron for low gain settings and an energy threshold at half the X-ray energy.

Another synchrotron with many different operating modes is SPring-8 in Japan (Fig. 7 ▶). As with the ESRF, it comprises continuous modes as well as modes with bunch separations of the order of the detector dead-time. Furthermore, two modes (mode *D* and *E*) exist in which a period of continuous beam is followed by a period of widely separated bunches. At high rates the continuous period can lead to saturation and voltage shifts in the shaper, which cause a dip in the observed rate.

Many synchrotrons, such as Diamond in the UK, only have continuous-like time structures leading to correction factors very similar to the SLS data shown above.

## 6. Conclusion

This article presents the results of a Monte Carlo simulation that is able to predict the rate correction factors for the PILATUS detector as a function of the detector settings and the time structure of the X-ray beam. The simulation has been successfully validated with data sets acquired at the Swiss Light Source and the Australian Synchrotron. The latter data show that an optimized time structure allows for measurements up to photon rates of 10^7^ photons s^−1^ pixel^−1^. The current PILATUS readout software corrects the observed count rate based on a paralysable detector model taking into account the dependency on the preamplifier gain settings (Kraft *et al.*, 2009 ▶). The integration of energy threshold and time-structure-dependent correction factors derived from the presented simulation will further improve the data quality at high photon fluxes. 

## Figures and Tables

**Table 1:** Principal properties of the PILATUS detector system

Pixel size	172 µm × 172 µm
Sensor material	Silicon
Sensor thickness	320 µm
Bias voltage	150 V
Readout chip design	0.25 µm CMOS
Counter depth	2^20^

**Table 2:** Experimental parameters of the SLS data set

X-ray energy (keV)	12, 16
Threshold-to-energy ratio	0.38, 0.50, 0.67
*Vrf* (V) (gain)	−0.2 (mid gain); −0.3 (low gain)
Bunch mode	Camshaft: 390 bunches separated by 2 ns, followed by a gap of 180 ns containing a single bunch
Number of analysed pixels	A few hundred

**Table 3:** Experimental parameters of the AS data set

X-ray energy (keV)	16
Threshold-to-energy ratio	0.50
*Vrf* (V) (gain)	−0.15 (high gain); −0.175, −0.2 (mid gain); −0.225, −0.25, −0.275, −0.3 (low gain)
Bunch mode	Default: 2 ns bunch separation with a current of trapezoidal form (Sobott *et al.*, 2012 ▶)
	Special: 180 ns and 240 ns bunch spacing
Number of analysed pixels	1
